# Anticholinesterase and Anti-Inflammatory Activities of the Essential Oils of Sawdust and Resin-Rich Bark from Azorean *Cryptomeria japonica* (Cupressaceae): In Vitro and In Silico Studies

**DOI:** 10.3390/ijms252212328

**Published:** 2024-11-17

**Authors:** Ana Lima, Filipe Arruda, Jorge Frias, Tanner Wortham, Alexandre Janeiro, Tânia Rodrigues, José Baptista, Elisabete Lima

**Affiliations:** 1 Institute of Agricultural and Environmental Research and Technology (IITAA), University of the Azores, 9700-042 Angra do Heroísmo, Portugal; ana.pr.lima@uac.pt (A.L.); filipe.mp.arruda@uac.pt (F.A.); alex-19961917@hotmail.com (A.J.); tanyamsrod@gmail.com (T.R.); jose.ab.baptista@uac.pt (J.B.); 2 Department of Physics, Chemistry and Engineering (DCFQE), Faculty of Science and Technology, University of the Azores, 9500-321 Ponta Delgada, Portugal; 3 Department of Biology (DB), Faculty of Science and Technology, University of the Azores, 9500-321 Ponta Delgada, Portugal; jorge.mv.frias@uac.pt; 4 Biotechnology Centre of Azores (CBA), University of the Azores, Terceira, Azores, 9700-042 Angra do Heroísmo, Portugal; 5 The Perfumery, 621 Park East Blvd, New Albany, IN 47150, USA; twortham@theperfumery.com

**Keywords:** *Cryptomeria japonica*, biomass residue valorization, essential oil, α-pinene, ferruginol, molecular docking, cholinesterase inhibition, anti-inflammatory activity, brine shrimp lethality, circular bioeconomy

## Abstract

Alzheimer’s disease (AD), a multifactorial neurodegenerative disorder characterized by severe cognitive impairment, affects millions of people worldwide. However, AD therapy remains limited and mainly symptomatic-focused, with acetylcholinesterase (AChE) inhibitors being the major available drugs. Thus, AD is considered by the WHO as a disorder of public health priority. Among several strategies that have been identified to combat AD, the use of natural multi-target drug ligands (MTDLs) appears to be a promising approach. In this context, we previously found that the essential oils (EOs), obtained via hydrodistillation, from Azorean *Cryptomeria japonica* sawdust (CJS) and resin-rich bark (CJRRB) were able to exert antioxidant activity via different mechanisms of action. Therefore, in the present work, these EOs were screened for their (i) in vitro anti-AChE and anti-butyrylcholinesterase (BChE) activities, evaluated by a modified Ellman’s assay; (ii) in vitro anti-inflammatory potential, using the albumin denaturation method; and (iii) toxicity against *Artemia salina*. The CJRRB–EO exhibited both anti-AChE and anti-BChE activities (IC_50_: 1935 and 600 µg/mL, respectively), whereas the CJS–EO only displayed anti-BChE activity, but it was 3.77-fold higher than that of the CJRRB–EO. Molecular docking suggested that α-pinene and ferruginol compounds contributed to the anti-AChE and anti-BChE activities, respectively. Moreover, the anti-inflammatory activity of the CJS–EO, the CJRRB–EO, and diclofenac was 51%, 70%, and 59% (at a concentration of only 2.21 μg/mL), respectively, with the latter two presenting comparable activity. Concerning the EOs’ potential toxicity, the CJRRB–EO exhibited a lower effect than the CJS–EO (LC_50_: 313 and 73 µg/mL, respectively). Overall, the EOs from C. *japonica* biomass residues, chiefly the CJRRB–EO, displayed antioxidant, anticholinesterase, and anti-inflammatory activities in a concentration-dependent manner. These properties demonstrate that these residues may be suitable natural MTDLs for AD complementary therapy when administered through aromatherapy, or, alternatively, could serve as low-cost sources of valuable ingredients, such as α-pinene.

## 1. Introduction

Alzheimer’s disease (AD) is the most common type of dementia. By 2030, the number of people with the disease is expected to rise to more than 70 million worldwide due to an increase in the aging population. Indeed, unless there is a breakthrough in treatment, nearly one in every 2–3 people over the age of 85 will have AD [1].

This disorder is a severe irreversible syndrome of the central nervous system (CNS), characterized by a slow and progressive decline in cognitive function and language ability. Major pathologic hallmarks noticed in AD are neurotoxic plaques of amyloid-beta (Aβ) protein, hyperphosphorylated tau (τ) neurofibrillary tangles, oxidative stress, remarkable neuroinflammation, and synaptic and neuronal dysfunction. The abnormal cholinergic activity is associated with the dysregulation of key acetylcholine-modulating enzymes, such as acetylcholinesterase (AChE) and butyrylcholinesterase (BChE). This dysregulation leads to a reduction of the brain neurotransmitter acetylcholine (ACh) levels, which plays a critical role in cognitive decline and memory impairment seen in AD [2,3].

Currently, the available drugs for AD are predominantly AChE and/or BChE inhibitors, such as donepezil, a selective AChE inhibitor, and rivastigmine, a dual inhibitor of both AChE and BChE. However, the efficacy of these drugs to combat AD is limited, as they may cause adverse side effects, due to its alkaloid origin, and fail to fully halt the progression of this complex disease. In fact, AD has a multifactorial etiology, involving several mechanisms which may work altogether through interaction between genetic, molecular, and cellular events. Thus, researchers currently have turned their attention to developing multi-target drug ligands (MTDLs) to simultaneously inhibit more than one of the pathological hallmarks of AD [2,4,5]. For instance, compounds that have activity against cholinesterases and anti-inflammatory properties are potential multi-target agents to combat AD. On top of that, the search for potential therapeutic drugs from traditional medical use experience has become a proven avenue for drug discovery [6,7].

Aromatherapy with essential oils (EOs) from aromatic plants has been employed for centuries to alleviate symptoms and enhance memory and cognition in dementia patients. The therapeutic benefits are primarily attributed to the volatile bioactive secondary metabolites present in EOs. The diverse, and often complex, chemical composition of EOs plays a key role in delivering a wide range of health-promoting effects (e.g., stress relief, antiviral, antimicrobial, antioxidant, anti-inflammatory, and anticancer properties), with applications in several fields worldwide, such as the medical, pharmaceutical, agricultural, cosmetic, and food industries [8,9].

The main chemical constituents of EOs include terpenes and terpenoids [8]. Due to their characteristics, namely their lipophilicity and low molecular weight, EOs are readily absorbed by the body in three main ways, whether through the respiratory system, the skin, and/or orally [10,11]. Furthermore, due to their low toxicity [12] and ability to easily access the CNS via inhalation or by crossing the blood–brain barrier (BBB), volatiles of EOs have been considered as promising MTDLs for AD therapies [5,6,9,11,13,14,15,16].

Essential oils from conifer trees, long-lived organisms with significant ecological and economic importance [17], are of particular interest. This is not only due to their rich content of terpenes and terpenoids [18,19,20] with neuroprotective properties [21], but also because they can be extracted from the biomass residues of the wood-cutting industry, such as those from *Cryptomeria japonica* (Thunb. ex L. f.) D. Don (Cupressaceae), the target species in this study. *Cryptomeria japonica* is, currently, the most important commercial forestry tree in the Azores, accounting for 60% of the total wood-producing forest area [22]. Consequently, the logging activities involving this species generate large amounts of biomass residues (e.g., foliage, cones, and bark), which are often left unattended, representing an environmental issue. Furthermore, the timber industry, particularly sawmills, produces tons of biomass waste. Each year, around 1.3 km^2^ of the *C. japonica* cultivation area is approved for harvesting, yielding approximately 100,000 m^3^ of wood. It is estimated that about 30% of this yield consists of by-products, such as sawdust and bark, without any or little commercial use [23].

The foliage of *C. japonica* is, by far, the plant part that has been most studied and utilized by wood producers for the extraction of EOs which can find various health-promoting applications, including aromatherapy [24]. The EOs from Japanese *C. japonica* leaves, particularly their diterpenes, have already been identified as strong inhibitors of AChE activity, suggesting their potential as effective therapeutic agents for AD [25].

As part of our continuing strategy for the valorization of the major Azorean *C. japonica* biomass residues that remain underutilized, we recently demonstrated that the EOs, obtained via hydrodistillation (HD), from Azorean *C. japonica* sawdust (CJS) and resin-rich bark (CJRRB) exhibit antioxidant activity (in a concentration-dependent manner), which is also an important feature in the management of AD. However, they displayed distinct antioxidant properties, which are linked to their unique chemical composition. Specifically, the CJS–EO, richer in sesquiterpenoids, demonstrated superior activity in the 2,2′-azinobis-3-ethylbenzothiazoline-6-sulfonic acid (ABTS) free-radical scavenging activity (FRSA) assay, while the CJRRB–EO, mainly constituted by monoterpenes, exhibited the best β-carotene-linoleic acid bleaching activity. However, both of the EOs showed similar activity in the 2,2-diphenyl-1-picrylhydrazyl (DPPH) FRSA assay, albeit weaker than those observed in the other referred assays [26].

In this context herein, we investigated the two aforementioned EO samples, in regard to their (i) in vitro anticholinesterase activities, evaluated by a modified Ellman’s assay; (ii) in vitro anti-inflammatory potential, using the inhibition of bovine serum albumin (BSA) denaturation assay; and (iii) toxic effects, assessed by an in vivo assay employing the nauplii of brine shrimp (*Artemia salina* Leach). In addition, molecular docking studies were performed to predict the possible EO compounds (EOCs) interacting within the active sites of AChE and BChE. Overall, the results of this study will contribute to an increase in knowledge of the potentialities of *C. japonica* biomass residues, particularly in the neuroscience field, which, in turn, can help *C. japonica*’s EO industry to meet different market demands and, consequently, contribute to the use of Azorean forestry biomass residues in a more economic and sustainable way.

## 2. Results and Discussion

### 2.1. Essential Oil Composition

The yield values of the CJS and CJRRB EO samples, obtained via the HD method, were 2.7 and 8 mL/kg on a dry weight (d.w.) basis, respectively, revealing a significant difference between the samples’ yields. The EOs’ chemical composition, as assessed in our earlier work [26], using GC/MS, also showed a different profile. Table 1 shows their major EOCs (≥ 3%) and the total percentage of the EOCs, grouped according to their chemical class, namely, monoterpene hydrocarbons (MH), oxygenated monoterpenes (OM), sesquiterpene hydrocarbons (SH), oxygenated sesquiterpenes (OS), diterpene hydrocarbons (DH), and oxygenated diterpenes (OD). The CJS–EO sample is richer in OS (67%), mainly β+α-eudesmol (13.5%) and 1-epicubenol (10.7%), and OD (15%), including ferruginol (3.6%), while the CJRRB–EO is predominantly composed of MH (64%), mainly α-pinene (43%), followed by limonene (8.9%) and δ-3-carene (6.0%). Curiously, MH and OM were not present in the CJS–EO sample.

### 2.2. Anticholinesterase Activity

The inhibition of cholinesterase activity has become a key part of therapeutic interventions, with traditional cholinesterase inhibitors often being naturally derived agents [2]. In fact, among the five drugs that have already been approved for the AD treatment, four are AChE inhibitors, namely tacrine (discontinued due to adverse effects), donepezil, rivastigmine, and galantamine [28]. Therefore, there is increasing scientific interest in the search for new anticholinesterase inhibitors from natural sources, including EOs, whose neuroprotective potential for age-related neurodegenerative diseases is widely evaluated worldwide [16].

**Figure 1:**
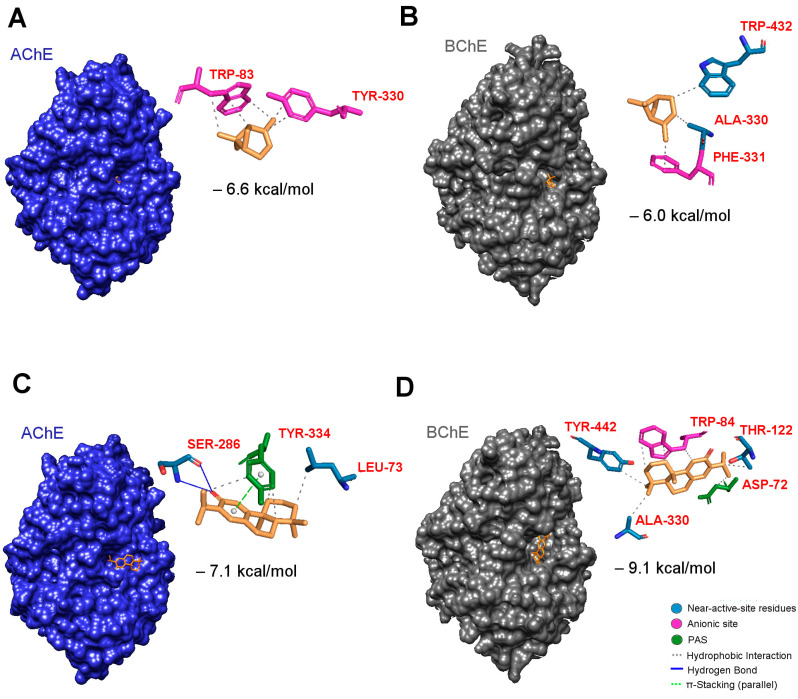
The docking simulations of both of the ligands (α-pinene and ferruginol) on the AChE (Blue) and BChE (Grey) enzymes, generated by AutoDock Vina: (**A**) The best binding poses of α-pinene with AChE; the interactions include hydrophobic interactions with W83 and Y330 from the anionic site (Mangenta). (**B**) The best binding poses of α-pinene with BChE; shows the presence of hydrophobic interactions with A330 and W432, along with F331 from the anionic site (Mangenta). (**C**) The best binding poses of ferruginol with AChE; the main interactions include hydrogen bonds with S286 and hydrophobic interactions with L73, along with π stacking and hydrophobic interactions with Y334 from the PAS site. (**D**) The best binding poses of ferruginol with BChE; all of the interactions include hydrophobic close contacts with W84 from the anionic site and D72 from the PAS site, along with several hydrophobic interactions with T122, A330, and Y442.

The evaluation of the anticholinesterase activity of the CJS and CJRRB EOs, as well as of α-pinene, was performed by using two key enzymes (AChE and BChE), and the results are presented in Table 2 as IC_50_ values. The tested EOs displayed inhibitory activities lower than that of the positive control (donepezil) in all of the enzymatic assays, yet the side effects associated with synthetic cholinesterase inhibitors are expected to differ from those of natural agents. Both of the EOs exhibited activity against BChE, but only the CJRRB–EO demonstrated activity against AChE, albeit weak, with an IC_50_ value of 1935 ± 338 µg/mL. On the contrary, the CJS–EO did not have any ability to inhibit AChE but displayed a remarkable BChE inhibition when compared to the CJRRB–EO (IC_50_: 159 ± 104 vs. 600 ± 238 µg/mL, respectively). Moreover, both of the EOs exhibited greater selectivity for BChE over AChE, whereas α-pinene showed a stronger affinity for AChE, similarly to the standard donepezil, as seen in Table 2. Indeed, as shown in Figure 1A,B, by molecular docking, the α-pinene compound showed a higher affinity towards AChE (−6.6 kcal/mol) compared to BChE (−6.0 kcal/mol). This higher selectivity of α-pinene for the AChE enzyme is due to the presence of five hydrophobic interactions with two amino acids (W83 and Y330) that are part of the anionic site, whereas in BChE, only one hydrophobic interaction occurs with F331 from the anionic site, and the other two hydrophobic interactions occur with residues near the active site.

Owokotomo et al. [29] also demonstrated that monoterpene-rich EOs (particularly α-pinene) exhibit a strong potential for inhibiting AChE in in vitro studies. It is well known that bicyclic monoterpenes, such as α-pinene and δ-3-carene, are potent AChE inhibitors [30,31]. In another work, molecular docking studies have demonstrated that these compounds, which are also the major constituents of the CJRRB–EO (43% and 6%, respectively, Table 1), interact with AChE through hydrophobic interactions [32] and exhibit a mixed-type mode of inhibition, like that of donepezil. This mechanism may partially explain the anti-AChE activity observed in the CJRRB–EO. In addition, the α-pinene-rich EO from the berries of *Juniperus communis* L. (Cupressaceae) exhibits the inhibition of AChE in Aβ(1–42)-treated rats [21]. Hence, the CJRRB–EO may present a sustainable resource for obtaining potent inhibitors of AChE activity. Alternatively, as both of the *C. japonica* EOs exhibit a greater selectivity for BChE over AChE, they offer distinct advantages, as the ratio of BChE/AChE activity can dramatically increase during the progression of AD [3]. Consequently, BChE is a proposed drug target for the treatment of the advanced stages of AD, where aromatherapy using *C. japonica* EOs may be especially beneficial. Furthermore, recent studies have revealed that inhibitors targeting BChE, or dual inhibitors of both cholinesterases, offer a more effective treatment for AD with fewer side effects compared to AChE-specific inhibitors [33].

On the other hand, the highest ferruginol content in the CJS–EO may explain its remarkable anti-BChE activity when compared to the CJRRB–EO. In fact, Bakir et al. [34] reported that this abietane-type diterpene exhibits higher anti-BChE activity than that of the standard galanthamine (inhibition values of 98.92% and 78.28% at 25 µg/mL concentration, respectively). Murata et al. [25] demonstrated that the EO from the leaves of Japanese *C. japonica* exhibits potent anti-AChE activity, which was mainly attributed to the presence of minor compounds, nezukol, and ferruginol, with IC_50_ values of 300 µM and 95 µM, respectively. Nezukol showed a mixed-type inhibition, while ferruginol showed a competitive-type inhibition, against AChE. However, the same study also revealed that these diterpenoids showed a greater selectivity for BChE over AChE, with IC_50_ values of 155 µM for nezukol and 22 µM for ferruginol, demonstrating a competitive mode of action. In fact, in our study, the molecular docking simulations of ferruginol in the active site of both enzymes, AChE and BChE, revealed a higher selectivity of ferruginol towards BChE, with a binding energy of −9.1 kcal/mol, and a lower affinity to AChE, presenting poses with a significantly lower binding energy of −7.1 kcal/mol (Figure 1C,D). The interaction profile of ferruginol/BChE (Figure 1D) explains the stronger interaction due to the presence of seven hydrophobic interactions. Four of them are with one amino acid (W84) from the anionic site and with another (D72) from the peripheral anionic site (PAS). On the other hand, the ferruginol/AChE binding pose (Figure 1C) presents a weaker interaction profile, with only three hydrophobic interactions and one π stacking interaction with the amino acid Y334 from the active site (PAS).

Another interesting work demonstrated that the Japanese *C. japonica* wood EO affects autonomic nervous activity, inducing anti-stress effects, in human studies [35]. Although, in the present study, the minor components of the CJS–EO may play an important role in the overall EO activity, it is sometimes unreliable to fractionate the mixture into its pure constituents, as the anti-AChE/BChE activity is often reduced, compared to the crude EO mixture [32,36].

### 2.3. Anti-Inflammatory Activity

Cholinesterase inhibitors are not able to completely stop the progression of AD, and, therefore, there is a need to develop MTDLs that can target several symptoms of AD, such as oxidative stress and inflammation [2]. The latter plays a key role in the pathogenesis of AD, triggering the overproduction of Aβ and τ proteins, which, in turn, induces an oxidative–inflammatory response, leading to a vicious cycle of neuroinflammation and pathology [9]. Hence, this study also highlighted the in vitro anti-inflammatory potential of the CJRRB and CJS EOs, as well as of α-pinene, using the anti-denaturation of BSA method. The results are summarized in Table 3.

The tested EOs, along with the reference drug, show a concentration-dependent inhibition of BSA against heat-induced denaturation, ranging from 2.21 to 17.71 μg/mL.

In particular, the present findings showed a percentage of inhibition of 51%, 70%, and 59% at a concentration of only 2.21 μg/mL, for the CJS–EO, the CJRRB–EO, and DCF (a commercial, non-steroidal, anti-inflammatory drug), respectively. Although the CJRRB–EO demonstrated the strongest anti-inflammatory effect, both of the EOs showed comparable activity to the standard DFC (Table 3). While α-pinene is the major component in the CJRRB–EO, the proportion of its enantiomers in the EO is unknown. In any case, (–)-α-pinene does not appear to be solely responsible for the observed anti-inflammatory activity in the CJRRB–EO. Essential oils are complex mixtures, and their biological effects often result from the synergistic interactions between both their major and minor components [8,37].

Although many studies have explored the anti-inflammatory properties of EOs, no research has been conducted specifically on the EOs of *C. japonica*. Nevertheless, Shyur el al. [38] demonstrated that terpenoids, among them sugiol, a phenolic abietane derivative of ferruginol, isolated from *C. japonica* wood extracts, exhibited potent anti-inflammatory and hepatoprotective activities in both in vitro and in vivo models. Their studies revealed that these compounds significantly reduced inflammation and protected liver cells, highlighting their potential as therapeutic agents for treating inflammatory and liver-related conditions.

Overall, both of the studied EOs had significant anti-inflammatory effects, supporting the view that aromatic plant extracts possess neuroprotective properties and considerable medicinal value for the treatment of AD. Moreover, in our previous study [26], it was demonstrated that both these EOs were able to exert antioxidant activity via different mechanisms of action. Therefore, the *C. japonica* EOs from biomass residues can also counteract free radical-mediated oxidative stress and thereby protect the brain from neuronal damage.

Moreover, monoterpene-rich EOs, particularly those abundant in α-pinene, from other conifers of the Cupressaceae family, have been shown to enhance the oxidative stress profile in AD rat models [21] and inhibit AChE activity in vitro [31]. Collectively, EOs and EOCs exhibit neuroprotective effects against Aβ-induced toxicity through mechanisms such as anti-amyloid, anti-inflammatory, and antioxidant actions. Sniffing volatile organic compounds could represent a novel approach to preventing or delaying early-stage cognitive impairment, offering a promising potential in the treatment of cognitive dysfunction [12].

### 2.4. Brine Shrimp Lethality Activity (BSLA)

The BSLA determination has been used for several purposes, including as an alternative to in vivo tests to screen the toxic potential of plant extracts in humans, as well as a potent tool for drug discovery from many extracts obtained from medicinal and aromatic plants [39,40].

The BSLA of the studied EO samples revealed that the CJS–EO was significantly more toxic than the CJRRB–EO, at 100 µg/mL concentration, with 68.0 ± 15% and 8.3 ± 4% mortality rate values (after 24 h exposure), respectively. In addition, the estimated lethal concentration 50 and 90 (LC_50_ and LC_90_) values of the studied EOs, along with their toxicity class, according to Karchesy’s criterion [41], are presented in Table 4.

The LC_50_ values (obtained after 24 h exposure) of CJS–EO and CJRRB–EO were 73 µg/mL and 313 µg/mL, respectively. According to Karchesy’s toxicity criterion [41], both of the EO samples were toxic (LC_50_ < 1000 μg/mL). However, the CJS–EO was considered strongly toxic to brine shrimp (LC_50_ < 100 μg/mL), while the CJRRB–EO was considered moderately toxic (LC_50_: 100–500 μg/mL). It is well documented that the LC_50_ from the brine shrimp toxicity test correlates with the LC_50_ value in animal models [42]. Nevertheless, the CJS–EO could have applications in other pharmacological areas, such as antitumor. In fact, EOs that were considered strongly toxic in the BSLA test have served as leads for further studies on bioactive compounds, such as ferruginol.

In summary, the CJRRB–EO exhibited lower toxicity against the brine shrimp compared to the CJS–EO. However, future work should involve the toxicological evaluation of these EOs on mammalian animal models.

## 3. Materials and Methods

### 3.1. Chemicals and Reagents

A standard mixture of C7-C33 *n*-alkanes was acquired from Restek (Bellefonte, PA, USA). Anhydrous sodium sulphate (Na_2_SO_4_), (–)-α-pinene (≥97%), sodium phosphate dibasic (Na_2_HPO_4_), sodium phosphate monobasic (NaH_2_PO_4_), acetylthiocholine iodide (ATChI), butyrylthiocholine iodide (BTChI), AChE from *Electrophorus electricus* L. (electric eel) and BChE from equine serum, BSA, and diclofenac sodium (DCF) were purchased from Sigma-Aldrich (St. Louis, MO, USA). Donepezil was obtained through Merck (Darmstadt, Germany). The 5,5′-Dithiobis(2-nitro-benzoic acid) (DTNB) was bought from TCI (Tokyo, Japan). Dimethyl sulfoxide (DMSO) was purchased from Riedel-de Häen (Aktiengesellschaft, Seelze, Germany). Deionized water was used in all of the experiments.

### 3.2. Plant Material

The CJRRB sample was collected from a wound on a *C. japonica* tree on São Miguel Island, in the Azores archipelago (Portugal), in 2023, as described by Lima et al. [26]. The CJRRB sample was shade-dried at room temperature (20 °C) and pulverized into powder using a mechanical grinder. The CJS sample, i.e., the woodmeal of *C. japonica*, was obtained from the local carpentry industry on São Miguel Island. The sample was air-dried at room temperature (20 °C) prior to EO extraction [26].

### 3.3. Essential Oil Extraction

The EOs from the Azorean CJRRB and CJS samples were obtained via HD through a Clevenger-type extractor, according to the European Pharmacopoeia [43], as detailed in Lima et al. [26]. Briefly, the ratio of the plant material sample to water was 1:10 g/mL, and the time of distillation was approximately 3 h, starting from the first distillate drop. The isolated EOs were dehydrated with anhydrous Na_2_SO_4_ and stored in sealed amber vials at 4 °C until further analysis. Each HD process was performed in triplicate. The EO yield (%, *v*/*w*) was calculated on a d.w. basis.

### 3.4. Essential Oil Composition Analysis

Gas chromatography–mass spectroscopy (GC–MS) analyses were carried out with a GCMS–QP2010 Ultra gas chromatograph–mass spectrometer (Shimadzu, Tokyo, Japan), using a ZB–5MSPlus capillary column (5% phenyl, 95% methyl siloxane) with a dimension of 60 m length, 0.25 mm i.d., and a film thickness of 0.25 μm (Phenomenex Inc., Torrance, CA, USA). The oven’s temperature increased from 50 °C to 260 °C at a rate of 2 °C/min and was then held for 5 min at the final temperature. The injector and detector temperatures were set at 260 °C, and the transfer line temperature was set at 300 °C. A volume of 0.1 μL of the EO sample dissolved in methylene chloride (0.1 g/mL) was injected for analysis at a split ratio of 24.4:1. Helium was used as the carrier gas, at a linear velocity of 36.3 cm/s. The mass spectra were recorded over the 40–400 atomic mass units (amu) range at 0.3 scans per second, with an ionization energy of 70 eV and an ion source temperature of 260 °C [26]. The identification of the EOCs was assigned by matching (i) their recorded mass spectra with the standard mass spectra from several libraries (a lab-made library and the FFNSC4.0, NIST11, and Wiley10 libraries) and (ii) their retention indices (RI), calculated according to ISO 7609 [44], relative to a homologous series of n-alkanes (C7–C33). The relative concentration of each EOC was quantified by integrating total ion current (TIC) chromatogram peaks without correction factors, as in our previous report [26].

### 3.5. In Vitro Anticholinesterase Assays

The AChE and BChE inhibitory activities of the CJS and CJRRB EOs, as well as of α-pinene, were measured in vitro according to the colorimetric method developed by Ellman et al. [45], with some modifications [46]. For both assays, the samples under study were dissolved in DMSO and diluted in deionized water until the desirable concentrations were achieved. The concentration of DMSO in the final reaction mixture was <0.2%. Donepezil was used as reference, at a stock concentration of *ca* 183 μg/mL. A control reaction was performed with deionized water instead of sample. The absorbance (Abs) was measured at 405 nm using a microplate reader (Thermo Scientific Multiskan FC, Waltham, MA, USA). Each assay was carried out in triplicate.

For the anti-AChE assay, 120 µL of EO or EOC solution (18.75–4800 µg/mL), 110 µL of sodium phosphate buffer (0.1 M, pH 8.0), and 10 μL of AChE enzyme solution containing 0.25 U/mL were mixed in a microwell plate and incubated for 5 min at room temperature. Then, 10 μL of a substrate mix solution (equal parts of ATChI 75 mM and DTNB 3 mM) was added, and the Abs was read after 8 min of incubation.

For the anti-BChE assay, 50 µL of the EO or EOC solution (1.8–909 µg/mL), 150 µL of the aforementioned buffer, and 10 μL of the BChE enzyme solution containing 0.44 U/mL were mixed in a microwell plate and incubated for 10 min at room temperature. Then, 10 μL of a substrate mix solution (equal parts of BTChI 11 mM and DTNB 5 mM) was added, and the Abs was read after 15 min of incubation.

The percentage inhibition of the AChE or BChE enzymes was calculated by comparison with the negative control, through Equation (1):

(1) $$Inhibition\;\left(\%\right)=1-\left(\frac{{Abs}_{sample}}{{Abs}_{control}}\right)\;\times 100$$

The results are expressed as the half-maximal inhibitory concentration (IC_50_), obtained from the dose-effect curves by linear regression.

### 3.6. Molecular Docking

To analyze the interactions between the ligands (α-pinene and ferruginol) and the AChE (4EY5) or BChE (4BDS) enzymes, molecular docking simulations were carried out using the AutoDock Vina software 1.1.2. version [47]. Before the simulations, both of the enzyme crystal structures were subjected to energy minimization with the YASARA2 force field with an implicit solvent, using the YASARA Energy Minimization Server [48]. The docking simulations were run with an energy range of 4 and an exhaustiveness of 64 to generate the top 10 docking poses for each ligand/AChE and ligand/BChE situation. The search spaces were defined as the enzyme active site encompassing the catalytic triad, the anionic site, the acyl pocket, the PAS, and the oxyanion hole, according to Bajda et al. [49]. Molecular representations were prepared using the PyMOL (The PyMOL Molecular Graphics System, Version 2.3.3 Schrödinger, LLC, Portland, OR, USA) and UCSF Chimera [50]. Enzyme-ligand interaction profiles were obtained using PLIP web tool [51].

### 3.7. Anti-Inflammatory Activity

The anti-inflammatory activity of the CJS and CJRRB EOs, as well as of α-pinene, was determined through an albumin denaturation inhibition assay, conducted as described by Matotoka et al. [52], with slight modifications to introduce a microplate for a higher throughput. A BSA solution (4%, *w*/*v*) was prepared in a phosphate buffered saline (PBS, pH 6.4). Stock solutions of each EO were mixed in DMSO at a concentration of 100 mg/mL and then diluted in PBS. Various concentrations of the test solutions (2.21–17.71 µg/mL) of the EOs were taken, each in a volume of 50 μL and mixed with 250 μL (4% *w*/*v* BSA). The product (negative) control solution (300  μL) consisted of 250 μL of the PBS and 50 μL of each EO solution, to consider the coloration of the EOs. Then, 250 μL of the BSA solution (4%) with 50 μL of the PBS were used as a test solution control, representing 100% protein denaturation. The solutions were then heated at 90 °C for 14 min in an oven and cooled for 15 min under laboratory conditions. The turbidity of the solutions (the level of protein precipitation) was measured at 650 nm in a microplate reader. The PBS was used as a blank. The results were compared with DCF as a standard.

The anti-inflammatory activity was calculated as a percentage of the inhibition of protein denaturation, using Equation (2):

(2) $$Denaturation\;inhibition\;\left(\%\right)=1-\left(\frac{{Abs}_{sample}}{{Abs}_{control}}\right)\;\times 100$$

### 3.8. Brine Shrimp Lethality Activity (BSLA) Assay

The toxicity potential of the EO samples was assessed by an in vivo assay employing the nauplii of brine shrimp. The cysts of brine shrimp (JBL GmbH & Co. KG, Neuhofen, Germany) were purchased locally and hatched in artificial seawater for 48 h.

The BSLA assay was performed according to a slightly modified Meyer et al. [40] method [22]. A stock solution of each EO was prepared by dissolving 150 mg of the sample in DMSO to a final volume of 0.5 mL. Then, the stock solution was diluted to 1 mg/mL in water and sonicated, which was then diluted to the range of 50–300 μg/mL in artificial seawater. Control samples of artificial seawater and DMSO (<0.1%, *v*/*v*) were also prepared to correct values with the natural mortality rate. In each well of a microplate, ten to fifteen nauplii were brought into contact with the EO, as well as with the control samples [22]. All of the assays were performed in triplicate. After 24 h of contact, the mortality percentage of the nauplii was calculated according to Abbott’s [53] formula (Equation (3)):

(3) $$Mortality\;\left(\%\right)=\left[\frac{\left(L_{control}-L_{sample}\right)}{L_{control}}\right]\times 100$$

where L is the living nauplii.

### 3.9. Statistical Analysis

All of the experiments were executed in triplicate, and the data are expressed as mean ± standard deviation (SD). A one-way analysis of variance (ANOVA) with a posthoc Tukey’s test was used to determine any significant statistical differences between the EOs and the standards, whereby the results were deemed significant at a *p*-value < 0.05. The LC_50_ and LC_90_ with 95% CI were determined by Probit Analysis, using the IBM SPSS Statistics version 27.0 software (SPSS Inc., Chicago, IL, USA).

## 4. Conclusions

There is growing interest in exploring the potential of EOs in neurodegenerative disease therapy due to their neuroprotective benefits, including antioxidant, anticholinesterase, and/or anti-inflammatory activities.

In this context, this is the first study on the anticholinesterase and anti-inflammatory activities of EOs from Azorean *C. japonica* sawdust and bark, which have been previously reported as sustainable sources for antioxidant compounds. Recent studies have revealed that inhibitors targeting BChE, or dual inhibitors of both cholinesterases (AChE and BChE), offer a more effective treatment for AD with fewer side effects compared to AChE-specific inhibitors. Thus, a natural product like the CJRRB–EO, which possesses dual cholinesterase inhibition, would be a promising therapeutic agent to combat AD. This EO has a high lipophilic content (64% of MH), mainly of α-pinene, which may facilitate faster cellular entry to exert its neuroprotective effects. Moreover, the CJRRB–EO exhibited the highest yield (*ca*. three times higher than that obtained from CJS), the lowest toxicity against the *A. salina* nauplii, and demonstrated an anti-inflammatory effect comparable to that of the standard diclofenac.

All these in vitro neuroprotective properties highlight the potential of *C. japonica* EOs as natural MTDLs for AD complementary therapy when administered through aromatherapy.

However, further investigations are necessary to confirm its biological targets (in vivo), as well as the potential human toxicity, before any recommendations can be made for its use as an anti-dementia treatment.

## Figures and Tables

**Table 1:** The composition of some major components (≥3%) of the essential oils (EOs), isolated via hydrodistillation of Azorean *Cryptomeria japonica* sawdust (CJS) and resin-rich bark (CJRRB) (data from Lima et al. [26]).

Component	Class	RT	RI_L_	RI_C_	Relative Content (%)	
					CJS–EO	CJRRB–EO
α-Pinene	MH	12.54	932	927		42.74
δ-3-Carene	MH	16.82	1008	1003		6.02
Limonene	MH	18.11	1024	1022		8.93
δ-Cadinene	SH	50.44	1522	1507	6.42	7.23
epi-Cubebol	OS	49.06	1493	1486	4.73	0.79
Cubebol	OS	50.26	1514	1504	6.76	1.28
1-Epicubenol	OS	56.87	1627	1615	10.74	1.93
τ-Cadinol	OS	57.71		1632	5.90	
δ-Cadinol	OS	57.93		1636	4.32	0.40
β+α-Eudesmol	OS	58.40	1649/1652	1643	13.54	0.54
β-Bisabolenal	OS	62.29	1768	1714	4.03	
Sandaracopimarinal	OD	84.53	2184	2164	3.03	0.09
Sandaracopimarinol	OD	88.30	2269	2253	5.48	
*trans*-Ferruginol	OD	90.37	2331	2301	3.64	0.90
**Identified components (%)**					95.71	96.92
**Grouped components (%)**						
Monoterpene hydrocarbons (MH)					0.00	63.97
Oxygenated monoterpenes (OM)					0.00	5.42
Sesquiterpene hydrocarbons (SH)					13.38	19.09
Oxygenated sesquiterpenes (OS)					66.64	6.39
Diterpene hydrocarbons (DH)					0.86	0.16
Oxygenated diterpenes (OD)					14.83	1.89

Legend: RI_L_—retention indices from the literature [27]; RI_C_—retention indices, calculated on a ZB–5MSPlus capillary column; RT—retention time (minutes) values, calculated on the same column.

**Table 2:** The cholinesterase inhibitory activity of the essential oils (EOs), isolated via hydrodistillation of Azorean *Cryptomeria japonica* sawdust and resin-rich bark and some of their components.

Samples	Cholinesterase Inhibitory Activity (IC_50_, µg/mL)	
	AChE	BChE
Sawdust EO	NA	159 ± 104 ^b^
Resin-rich bark EO	1935 ± 338 ^c^	600 ± 238 ^c^
(–)-α-Pinene	96 ± 4 ^b^	648 ± 22 ^c^
Ferruginol	27.3 *	6.3 *
Donepezil	0.01 ± 0.00 ^a^	1.85 ± 0.29 ^a^

Values are mean ± SD (*n* = 3). Different letters in the same column indicate statistically significant differences (*p* < 0.05). Legend: IC_50_—half-maximal inhibitory concentration; AChE—acetylcholinesterase; BChE—butyrylcholinesterase; NA—no activity; * Data retrieved from [25].

**Table 3:** The anti-inflammatory effect of the essential oils (EOs), isolated via hydrodistillation of Azorean *Cryptomeria japonica* sawdust and resin-rich bark in the denaturation of bovine serum albumin inhibition assay.

Samples	Protection Against Protein Denaturation (%)			
	2.21 µg/mL	4.43 µg/mL	8.85 µg/mL	17.71 µg/mL
Sawdust EO	51 ± 9 ^bc^	67 ± 15 ^ab^	75 ± 18 ^ab^	84 ± 15 ^ab^
Resin-rich bark EO	70 ± 3 ^a^	86 ± 7 ^a^	89 ± 9 ^a^	92 ± 3 ^a^
(–)-α-Pinene	45 ± 6 ^c^	62 ± 6 ^b^	70 ± 8 ^b^	77 ± 12 ^b^
DCF	59 ± 10 ^ab^	76 ± 11 ^ab^	84 ± 12 ^ab^	87 ± 14 ^ab^

Values are mean ± SD (*n* = 3). Different letters in the same column indicate statistically significant differences (*p* < 0.05). Legend: DCF—diclofenac sodium.

**Table 4:** Estimated values of lethal concentration 50 and 90 (LC_50_ and LC_90_) of essential oils (EOs) isolated via hydrodistillation of Azorean *Cryptomeria japonica* sawdust and resin-rich bark against *Artemia salina* nauplii after 24 h of exposure.

Samples	Concentration (µg/mL)	LC_50_	LC_90_	Intercept ± SEM	Slope ± SEM	Toxicity Class *
		(95% CI)	(95% CI)		(95% CI)	
Sawdust EO	50, 100, 150, 300	73 ^a^ (68–79)	136 ^a^ (122–159)	−9 ± 1 ^a^	4.8 ± 0.5 ^a^ (3.8–5.8)	Strongly toxic
Resin-rich bark EO	50, 100, 150, 300	313 ^b^ (268–402)	882 ^b^ (611–1735)	−8 ± 1.9 ^a^	3.2 ± 0.9 ^a^ (1.5–4.9)	Moderately toxic

LC values, intercepts, and slopes within the same column followed by the same letter are not significantly different. Legend: LC_50_ and LC_90—_the concentration required to kill 50% and 90% of the population of *A. salina* nauplii, respectively; CI—confidence interval; SEM—standard error mean; * toxicity classification based on the LC_50_ value against *A. salina*, according to Karchesy’s criterion [41].

## Data Availability

The data are contained within the article.

## Abbreviations

Aβ, amyloid-beta; τ, tau; Abs, absorbance; ABTS, 2,2′-azinobis-3-ethylbenzothiazoline-6-sulfonic acid; ACh, acetylcholine; AChE, acetylcholinesterase; AD, Alzheimer’s disease; BBB, blood–brain barrier; BChE, butyrylcholinesterase; BSA, bovine serum albumin; BSLA, brine shrimp lethality activity; CJRRB, *Cryptomeria japonica* resin-rich bark; CJS, *Cryptomeria japonica* sawdust; CNS, central nervous system; DCF, diclofenac sodium; DH, diterpene hydrocarbons; DPPH, 2,2-diphenyl-1-picrylhydrazyl; d.w., dry weight; EO, essential oil; EOC, essential oil component; FRSA, free radical-scavenging activity; GC–MS, gas chromatography–mass spectroscopy; HD, hydrodistillation; IC_50_, half-maximal inhibitory concentration; MH, monoterpene hydrocarbons; MTDLs, multi-target directed ligands; OD, oxygenated diterpenes; OM, oxygenated monoterpenes; OS, oxygenated sesquiterpenes; PAS, peripheral anionic site; RI, retention indices; SH, sesquiterpene hydrocarbons.

## References

[1] McDade E., Bateman R.J. (2017). Stop Alzheimer’s before it starts. Nature.

[2] Sharma K. (2019). Cholinesterase inhibitors as Alzheimer’s therapeutics. Mol. Med. Rep..

[3] Chen W.N., Chin K.W., Tang K.S., Agatonovic-Kustrin S., Yeong K.Y. (2023). Neuroprotective, neurite enhancing, and cholinesterase inhibitory effects of Lamiaceae family essential oils in Alzheimer’s disease model. J. Herb. Med..

[4] Wang T., Liu X.H., Guan J., Ge S., Wu M.B., Lin J.P. (2019). Advancement of multi-target drug discoveries and promising applications in the field of Alzheimer’s disease. Eur. J. Med. Chem..

[5] Lima E., Medeiros J. (2024). Terpenes as potential Anti-Alzheimer’s disease agents. Appl. Sci..

[6] Wang Y., Huang L.Q., Tang X.C., Zhang H.Y. (2010). Retrospect and prospect of active principles from Chinese herbs in the treatment of dementia. Acta Pharmacol. Sin..

[7] Chen X., Drew J., Berney W., Lei W. (2021). Neuroprotective natural products for Alzheimer’s Disease. Cells.

[8] Bakkali F., Averbeck S., Averbeck D., Idaomar M. (2008). Biological effects of essential oils—A review. Food Chem. Toxicol..

[9] Ma Y., Li Y., Yin R., Guo P., Lei N., Li G., Xiong L., Xie Y. (2023). Therapeutic potential of aromatic plant extracts in Alzheimer’s disease: Comprehensive review of their underlying mechanisms. CNS Neurosci. Ther..

[10] Silva H. (2020). A descriptive overview of the medical uses given to Mentha aromatic herbs throughout history. Biology.

[11] Vora L.K., Gholap A.D., Hatvate N.T., Naren P., Khan S., Chavda V.P., Balar P.C., Gandhi J., Khatri D.K. (2024). Essential Oils for clinical aromatherapy: A comprehensive review. J. Ethnopharmacol..

[12] Shi A., Long Y., Ma Y., Yu S., Li D., Deng J., Wen J., Li X., Wu Y., He X. (2023). Natural essential oils derived from herbal medicines: A promising therapy strategy for treating cognitive impairment. Front. Aging Neurosci..

[13] Maggio A., Rosselli S., Bruno M. (2016). Essential Oils and Pure Volatile Compounds as Potential Drugs in Alzheimer’s Disease Therapy: An Updated Review of the Literature. Curr. Pharm. Des..

[14] Cho K.S., Lim Y., Lee K., Lee J., Lee J.H., Lee I.-S. (2017). Terpenes from forests and human health. Toxicol. Res..

[15] Ball E.L., Owen-Booth B., Gray A., Shenkin S.D., Hewitt J., McCleery J. (2020). Aromatherapy for dementia. Cochrane Database Syst. Rev..

[16] Abd Rashed A., Abd Rahman A.Z., Rathi D.N.G. (2021). Essential oils as a potential neuroprotective remedy for age-related neurodegenerative diseases: A Review. Molecules.

[17] Bhardwaj K., Islam M.T., Jayasena V., Sharma B., Sharma S., Sharma P., Kuča K., Bhardwaj P. (2020). Review on essential oils, chemical composition, extraction, and utilization of some conifers in Northwestern Himalayas. Phyther. Res..

[18] Bhardwaj K., Silva A.S., Atanassova M., Sharma R., Nepovimova E., Musilek K., Sharma R., Alghuthaymi M.A., Dhanjal D.S., Nicoletti M. (2021). Conifers phytochemicals: A valuable forest with therapeutic potential. Molecules.

[19] Kopaczyk J., Wargula J., Jelonek T. (2020). The variability of terpenes in conifers under developmental and environmental stimuli. Environ. Exp. Bot..

[20] Mediavilla I., Guillamón E., Ruiz A., Esteban L.S. (2021). Essential oils from residual foliage of forest tree and shrub species: Yield and antioxidant capacity. Molecules.

[21] Cioanca O., Hancianu M., Mihasan M., Hritcu L. (2015). Anti-acetylcholinesterase and antioxidant activities of inhaled *Juniper* oil on amyloid beta (1–42)-induced oxidative stress in the rat hippocampus. Neurochem. Res..

[22] Janeiro A., Lima A., Arruda F., Wortham T., Rodrigues T., Baptista J., Lima E. (2024). Variations in essential oil biological activities of female cones at different developmental stages from Azorean *Cryptomeria japonica* (Thunb. ex L.f.) D. Don (Cupressaceae). Separations.

[23] Simas F.P.C. (2016). Assessment of the Potential as Fuel of the Main Forest Species in São Miguel Island, Azores. Master’s Thesis.

[24] Lima A., Arruda F., Medeiros J., Baptista J., Madruga J., Lima E. (2021). Variations in essential oil chemical composition and biological activities of *Cryptomeria japonica* (Thunb. ex L.f.) D. Don from different geographical origins: A critical review. Appl. Sci..

[25] Murata K., Tanaka K., Akiyama R., Noro I., Nishio A., Nakagawa S., Matsumura S., Matsuda H. (2018). Anti-cholinesterase activity of crude drugs selected from the ingredients of incense sticks and heartwood of *Chamaecyparis obtusa*. Nat. Prod. Commun..

[26] Lima A., Arruda F., Wortham T., Janeiro A., Rodrigues T., Baptista J., Lima E. (2024). Chemical compositions and in vitro antioxidant activities of the essential oils of sawdust and resin-rich bark from Azorean *Cryptomeria japonica* (Cupressaceae). Antioxidants.

[27] Adams R.P. (2007). Identification of Essential Oils by Gas Chromatography/Mass Spectrometry.

[28] Martins M., Silva R.M.M., Pinto M., Sousa E. (2020). Marine natural products, multitarget therapy and repurposed agents in Alzheimer’s disease. Pharmaceuticals.

[29] Owokotomo I.A., Ekundayo O., Abayomi T.G., Chukwuka A.V. (2015). In-vitro anti-cholinesterase activity of essential oil from four tropical medicinal plants. Toxicol. Rep..

[30] Miyazawa M., Yamafuji C. (2005). Inhibition of acetylcholinesterase activity by bicyclic monoterpenoids. J. Agric. Food Chem..

[31] Aazza S., Lyoussi B., Miguel M.G. (2011). Antioxidant and antiacetylcholinesterase activities of some commercial essential oils and their major compounds. Molecules.

[32] Chen S.X., Xiang J.Y., Han J.X., Yang-Feng, Li H.Z., Chen H., Xu M. (2022). Essential oils from spices inhibit cholinesterase activity and improve behavioral disorder in AlCl_3_ induced dementia. Chem. Biodivers..

[33] Zhou S., Huang G. (2022). The biological activities of butyrylcholinesterase inhibitors. Biomed. Pharmacother..

[34] Bakir D., Akdeniz M., Ertas A., Yilmaz M.A., Yener I., Firat M., Kolak U. (2020). A GC-MS method validation for quantitative investigation of some chemical markers in *Salvia hypargeia* Fisch. & C.A. Mey. of Turkey: Enzyme inhibitory potential of ferruginol. J. Food Biochem..

[35] Matsubara E., Tsunetsugu Y., Ohira T., Sugiyama M. (2017). Essential oil of Japanese cedar (*Cryptomeria japonica*) wood increases salivary dehydroepiandrosterone sulfate levels after monotonous work. Int. J. Environ. Res. Public Health.

[36] Orhan I., Kartal M., Kan Y., Sener B. (2008). Activity of essential oils and individual components against acetyl- and butyrylcholinesterase. Z. Naturforsch. C.

[37] Sousa D.P., Damasceno R.O.S., Amorati R., Elshabrawy H.A., de Castro R.D., Bezerra D.P., Nunes V.R.V., Gomes R.C., Lima T.C. (2023). Essential oils: Chemistry and pharmacological activities. Biomolecules.

[38] Shyur L.F., Huang C.C., Lo C.P., Chiu C.Y., Chen Y.P., Wang S.Y., Chang S.T. (2008). Hepatoprotective phytocompounds from *Cryptomeria japonica* are potent modulators of inflammatory mediators. Phytochemistry.

[39] Azra M.N., Noor M.I.M., Burlakovs J., Abdullah M.F., Abd Latif Z., Yik Sung Y. (2022). Trends and new developments in *Artemia* research. Animals.

[40] Meyer B.N., Ferrigni N.R., Putnam J.E., Jacobsen L.B., Nichols D.E., McLaughlin J.L. (1982). Brine shrimp: A conveninent general bioassay for active plant constituents. Planta Med..

[41] Karchesy Y.M., Kelsey R.G., Constantine G., Karchesy J.J. (2016). Biological screening of selected Pacific northwest forest plants using the brine shrimp (*Artemia salina*) toxicity bioassay. Springerplus.

[42] Parra A.L., Yhebra R.S., Sardiñas I.G., Buela L.I. (2001). Comparative study of the assay of *Artemia salina* L. and the estimate of the medium lethal dose (LD50 value) in mice, to determine oral acute toxicity of plant extracts. Phytomedicine.

[43] Council of Europe (2010). European Directorate for the Quality of Medicines, in European Pharmacopoeia.

[44] (1985). Essential Oils—Analysis by Gas Chromatography on Capillary Columns—General Method.

[45] Ellman G.L., Courtney K.D., Andres V., Featherstone R.M. (1961). A new and rapid colorimetric determination of acetylcholinesterase activity. Biochem. Pharmacol..

[46] Majid H., Silva F.V.M. (2020). Inhibition of enzymes important for Alzheimer’s disease by antioxidant extracts prepared from 15 New Zealand medicinal trees and bushes. J. R. Soc. N. Z..

[47] Trott O., Olson A.J. (2010). AutoDock Vina: Improving the speed and accuracy of docking with a new scoring function, efficient optimization, and multithreading. J. Comput. Chem..

[48] Krieger E., Joo K., Lee J., Lee J., Raman S., Thompson J., Tyka M., Baker D., Karplus K. (2009). Improving physical realism, stereochemistry, and side-chain accuracy in homology modeling: Four approaches that performed well in casp8. Proteins.

[49] Bajda M., Więckowska A., Hebda M., Guzior N., Sotriffer C.A., Malawska B. (2013). Structure-Based Search for New Inhibitors of Cholinesterases. Int. J. Mol. Sci..

[50] Pettersen E.F., Goddard T.D., Huang C.C., Couch G.S., Greenblatt D.M., Meng E.C., Ferrin T.E. (2004). UCSF Chimera—A visualization system for exploratory research and analysis. J. Comput. Chem..

[51] Adasme M.F., Linnemann K.L., Bolz S.N., Kaiser F., Salentin S., Haupt V.J., Schroeder M. (2021). PLIP 2021: Expanding the scope of the protein–ligand interaction profiler to DNA and RNA. Nucleic Acids Res..

[52] Matotoka M.M., Mashabela G.T., Masoko P. (2023). Phytochemical content, antibacterial activity, and antioxidant, anti-inflammatory, and cytotoxic effects of traditional medicinal plants against respiratory tract bacterial pathogens. Evid. Based Complement. Altern. Med..

[53] Abbott W.S. (1925). A method of computing the effectiveness of an insecticide. J. Econ. Entomol..

